# Responses to depressive symptom items exhibit a common mathematical pattern across the European populations

**DOI:** 10.1038/s41598-019-51499-w

**Published:** 2019-10-17

**Authors:** Shinichiro Tomitaka, Yohei Kawasaki, Kazuki Ide, Maiko Akutagawa, Yutaka Ono, Toshi A. Furukawa

**Affiliations:** 1 Department of Mental Health, Panasonic Health Center, Tokyo, Japan; 2 Department of Health Promotion and Human Behavior Department of Clinical Epidemiology, Kyoto University Graduate School of Medicine, Kyoto, Japan; 3 Clinical Research Center, Chiba University Hospital, Chiba, Japan; 4 Department of Pharmacoepidemiology, Graduate School of Medicine and Public Health, Kyoto University, Kyoto, Japan; 5 Center for the Promotion of Interdisciplinary Education and Research, Kyoto University, Kyoto, Japan; 6 Department of Drug Evaluation and Informatics School of Pharmaceutical Sciences, University of Shizuoka, Shizuoka, Japan; 7 Center for the Development of Cognitive Behavior Therapy Training, Tokyo, Japan

**Keywords:** Depression, Epidemiology

## Abstract

The theoretical distribution of responses to depressive symptom items in a general population remains unknown. Recent studies have shown that responses to depressive symptom items follow the same pattern in the US and Japanese populations, but the degree to which these findings can be generalized to other countries is unknown. The purpose of this study was to conduct a pattern analysis on the EU population’s responses to depressive symptom items using data from the Eurobarometer. The Eurobarometer questionnaires include six depressive symptom items from the 12-item General Health Questionnaire. The pattern analysis revealed that, across the entire EU population, the ratios between “score = 2” and “score = 1” and between “score = 3” to “score = 2” were similar among the six items and resulted in a common pattern. This common pattern was characterized by an intersection at a single point between “score = 0” and “score = 1” and a parallel pattern between “score = 1” and “score = 3” on a logarithmic scale. Country-by-country analyses revealed that the item responses followed a common characteristic pattern across all 15 countries. Our results suggest that responses to depressive symptom items in a general population follow the same characteristic pattern regardless of the specific country.

## Introduction

According to the World Health Organization, depression is the leading cause of disability worldwide, with over 300 million people affected^1^. As the diagnosis of depression is based on the severity of depressive symptoms, a variety of screening scales for depression have been developed to measure the level of symptom severity^2–4^. Extensive research has been conducted on the psychometric properties of depression screening scales^5–8^. However, the theoretical distribution of item responses to depressive symptom items in a general population, which is a fundamental research question, remains unaddressed.

This question is important for three main reasons. First, if the pattern of responses to depressive symptom items is established, it will contribute to a better understanding of how the severity of depressive symptoms is distributed in the general population. Second, statistical procedures that assume normality are currently used to evaluate the psychometrics of depression screening scales^9^. However, if the observed distributions of item responses considerably differ from the normal distribution, such statistical procedures would require consideration. Finally, if the responses to depressive symptom items are found to follow a specific mathematical pattern, it will provide insight into the characteristics of depressive symptoms.

**Figure 1:**
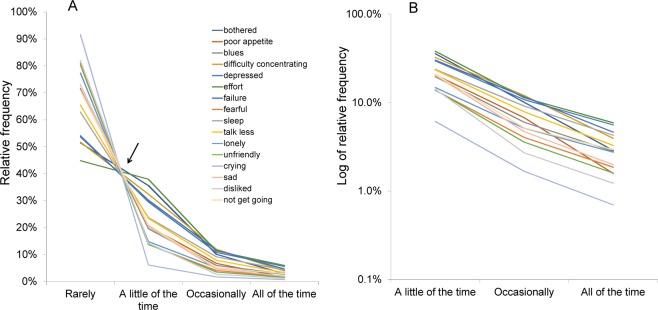
Responses to the 16 depressive symptom items are displayed using a normal scale (**A**) and a logarithmic scale (**B**). (**A**) The lines indicating the 16 items cross at a common area (black arrow) between “rarely” and “a little of the time.” From “a little of the time” to “all of the time,” the same lines display a decreasing pattern. (**B**) The lines for the 16 items exhibit a parallel pattern from “a little of the time” to “all of the time” on a logarithmic scale. Image credit: PLoS ONE, https://doi.org/10.1371/journal.pone.0165928.g001.

Recently, based on data from the Japanese Active Survey of Health and Welfare (32,000 individuals), we reported that item responses to the Center for Epidemiologic Studies Depression Scale (CES-D) followed a common pattern across the 16 depressive symptom items^10,11^. As illustrated in Fig. 1A, the lines indicating the responses to the 16 depressive symptom items cross at a common area between “rarely (score = 0)” and “a little of the time (score = 1)” (black arrow). From “a little of the time (score = 1)” to “all of the time (score = 3),” the lines display a decreasing pattern (Fig. 1A). On a logarithmic scale, the item response lines exhibit a parallel pattern from “a little of the time” to “all of the time” (Fig. 1B)^10,11^. Moreover, it was revealed that the similarity of the ratios between “score = 2” and “score = 1” and “score = 3” to “score = 2” in all 16 items resulted in a characteristic pattern^10,11^. It is important to note that the item response lines did not show this common pattern for the four CES-D positive affect items.

**Figure 2:**
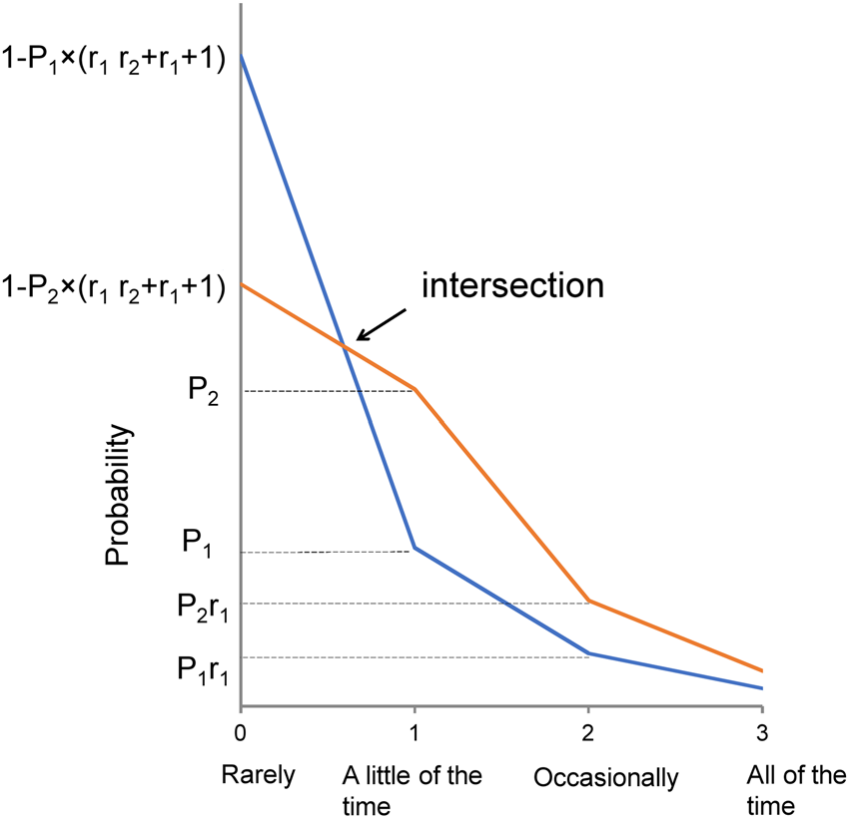
This mathematical model is based on the assumptions that the decreasing ratios of “occasionally” to “a little of the time” and “all of the time” to “occasionally” are constant for all items. If the probability of “score = 1” is presented as P*i* (*i* = item number) and the ratio of “score = 2” to “score = 1” and “score = 3” to “score = 2” are presented as two constants, r_1_ and r_2_, the probabilities of “score = 1,” “score = 2,” “score = 3,” and “score = 0” are expressed as P*i*, P*i*r_1_, P*i*r_1_r_2_, 1 – P*i* × (1 + r1 + r_1_r^2^), respectively.

Based on these findings, we proposed a model for responses to depressive symptom items on a Likert scale (0–3)^10,11^. As shown in Fig. 2, when the probability of “score = 1” is presented as P_*i*_ (*i* = item number) and the ratio of “score = 2” to “score = 1” and “score = 3” to “score = 2” are presented as two constants, r_1_ and r_2_, the probabilities of “score = 1,” “score = 2,” “score = 3,” and “score = 0” are expressed as P_i_, P_i_r_1_, P_i_r_1_r_2_, 1 – P_i × _(1 + r_1 + _r_1_r_2_), respectively. Previous studies have mathematically shown that, if the two constants r_1_ and r_2_ are the same for all items, all lines for the items cross at a single point between “score = 0” and “score = 1,” and the lines for the six items follow a parallel pattern from “score = 1” to “score = 3” on a logarithmic scale^10,11^.

Since the above studies are the first to report that responses to depressive symptom items follow such a model for all items in the general population, we carefully confirmed the reproducibility of this finding. As a result, the characteristic pattern of the item response lines has been replicated for the Patient Health Questionnaire (PHQ) data from the National Health and Nutrition Examination Survey and the Behavioral Risk Factor Surveillance Survey in the United States^12,13^, the Kessler Psychological Distress Scale data from the National Survey of Midlife Development in the United States^14^, and the CES-D data from the Irish Longitudinal Study on Ageing (TILDA)^15^. However, except for the TILDA, much of the research on the response patterns to these items has been confined to data from the US and Japanese populations. The degree to which these findings can be generalized to populations of other countries is unknown and, therefore, warrants an investigation.

The Eurobarometer is a series of public surveys that explore social issues pertaining to the European Union (EU). The 12-item General Health Questionnaire (GHQ-12) is a useful screening tool for detecting common mental disorders such as depression and anxiety disorder^16–18^. The GHQ-12 consists of six positive and negative items, and its six negative items were included in the Eurobarometer 56.1: Social Exclusion and Modernization of Pension Systems conducted in 2001^19^. It should be noted that, although the GHQ-12 is often labeled as a psychological distress scale, its six negative items are similar to the depressive symptom items on the CES-D and the PHQ-9^4,17,20^. Moreover, these items that overlap with the CES-D (e.g., “insomnia,” “depressed,” and “my life had been a failure”) and the PHQ-9 (e.g., “sleep disturbance,” “depressed,” “fidgety,” and “feeling bad about yourself”) exhibited the same characteristic item response pattern as the one identified in previous studies (Fig. 1)^10,13^. These findings indicate the possibility that responses to the negative GHQ-12 items also exhibit the same characteristic pattern as those in the CES-D and the PHQ-9. However, little is known about the mathematical pattern of the item responses to the six negative GHQ-12 items in a general population.

The Eurobarometer data provide a large sample size, which is a significant advantage for this study^19^. Generally, with a large sample size, an empirical distribution more closely approximates a theoretical distribution^21^. We analyzed the Eurobarometer data to investigate whether responses to the six negative GHQ-12 items exhibit the same characteristic pattern across all 15 EU populations.

It is important to note that, although item response theory (IRT) has become popular in current psychology and psychiatry research^22^, the present study was not based on IRT. IRT, which uses a model of the relationship between a latent trait and the probability of each response option, can calculate the parameters of the model^23^. The aim of the present study, however, was to inductively explore the mathematical pattern of the responses to depressive symptom items.

## Results

### Item responses in the entire EU population

Across the entire EU population, the responses to the six negative items exhibited the same pattern — the highest frequency for “not at all = 0,” and a decreasing pattern thereafter as item scores increased, with the lowest frequency observed for “much more than usual = 3” (Table 1). The ratios of “score = 2” to “score = 1” (mean ± SD = 0.35 ± 0.05) and “score = 3” to “score = 2” (mean ± SD = 0.29 ± 0.04) were stable to some extent among the six items. Of note, the similarity of these ratios across all six items results in the characteristic pattern of item responses^10^. The average ratio of “score = 2” to “score = 1” (0.35) was close to that of “score = 3” to “score = 2” (0.29).

**Table 1:** Responses to negative items in the GHQ-12 for the entire EU.

No	Item	Item response				Rate of “2” to “1”	Rate of “3” to “2”
		0	1	2	3		
1	Lost much sleep over worry	43.0%	37.3%	14.7%	5.0%	0.39	0.34
2	Unhappy and depressed	47.3%	35.8%	13.1%	3.7%	0.37	0.28
3	Losing confidence in yourself	61.7%	27.5%	8.4%	2.4%	0.30	0.28
4	Could not overcome your difficulties	53.1%	32.4%	11.7%	2.8%	0.36	0.24
5	Constantly under strain	47.8%	35.1%	13.5%	3.6%	0.38	0.26
6	Thinking of yourself as a worthless person	71.2%	21.4%	5.6%	1.9%	0.26	0.34
	Average	54.0%	31.6%	11.2%	3.2%	0.35 ± 0.05	0.29 ± 0.04

*Note*. Each of the six items is rated with one of the four response options: 0 (*not at all*), 1 (*no more than usual*), 2 (*rather more than usual*), and 3 (*much more than usual*). Average proportions of responses are presented as mean plus or minus one standard deviation.

**Figure 3:**
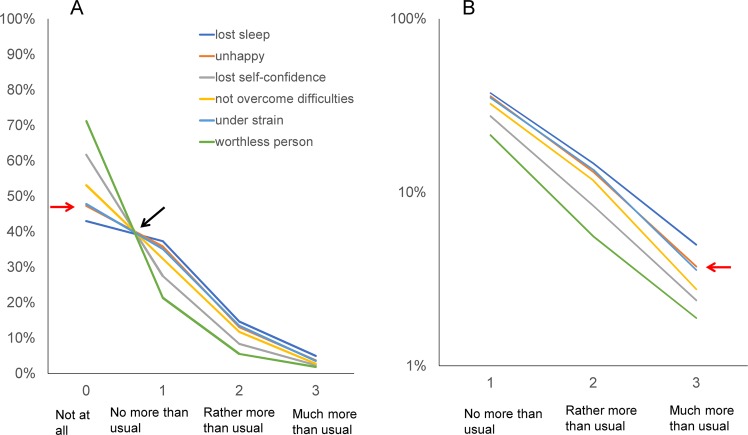
Responses to the six negative items are presented using a normal scale (**A**) and a logarithmic scale (**B**). (**A**) The lines for the six negative items cross at a common area (black arrow) between “score = 0” and “score = 1.” From “score = 1” to “score = 3,” the same lines display a decreasing pattern. (**B**) The lines for the six negative items exhibit a parallel pattern from “score = 1” to “score = 3” on a logarithmic scale.

To demonstrate the pattern of item responses, all lines of item responses were plotted on a single graph (Fig. 3). The graph displaying the six-item responses appeared as five lines because the two lines indicating “unhappy and depressed” and “constantly under strain” are close (red arrow). In fact, the probabilities of each of the four response options were very similar for “unhappy and depressed” and “constantly under strain” (Table 1).

All item response lines showed the same pattern across the six items (Fig. 3A), which is similar to the findings of previous studies (Fig. 1)^10^. As indicated by the black arrow in Fig. 3A, all item response lines intersected at a single point between “score = 0” and “score = 1” and displayed a decreasing pattern from “score = 1” to “score = 3.”

Using a logarithmic scale, the item response lines showed a parallel pattern between “score = 1” and “score = 3” (Fig. 3B). The two lines for “unhappy and depressed” and “constantly under strain” also intersected (red arrow). The item response lines between “score = 1” and “score = 3” appeared to follow a linear pattern without apparent fluctuations on a logarithmic scale, which is in line with the findings that the average ratio of “score = 2” to “score = 1” (mean = 0.35) was close to that of “score = 3” to “score = 2” (mean = 0.29) (Table 1).

### Item responses by country

**Figure 4:**
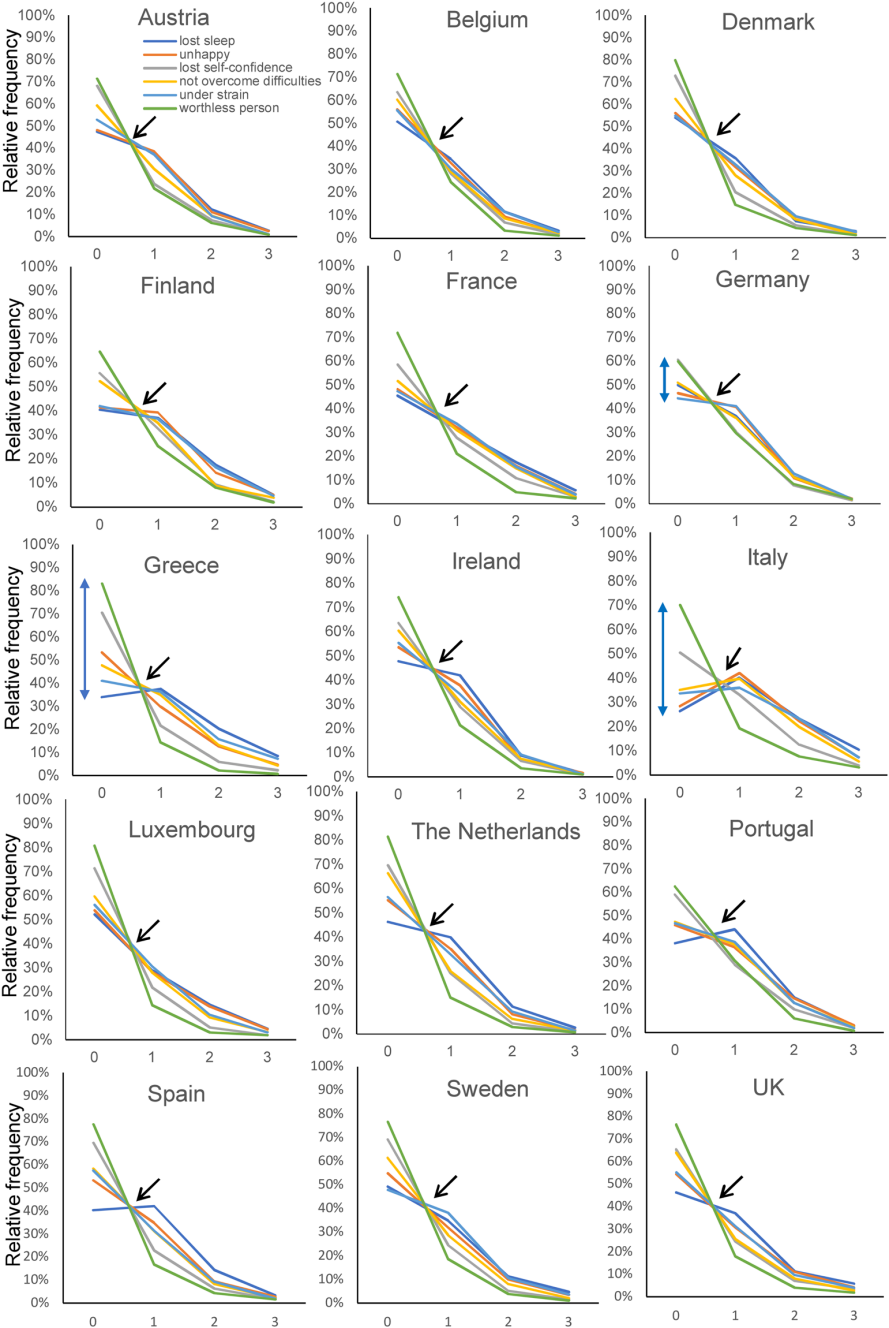
The responses to the negative items show a common pattern across the 15 European countries. The lines for the six negative items cross at a common area (black arrow) between “score = 0” and “score = 1.” From “score = 1” to “score = 3,” the same lines display a decreasing pattern.

Detailed information about the item responses for each European country is presented as Supplementary Data (Data set 1). Of note, the ratios of “score = 2” to “score = 1” and “score = 3” to “score = 2” were stable to some extent among the six items across the 15 EU countries. As shown in Fig. 4, the responses to the six items showed a common pattern across the 15 EU countries. As indicated by the black arrows, the lines for the six items intersected around a single point between “score = 0” and “score = 1,” after which they displayed a decreasing pattern across all 15 EU countries. Similar to the results of the entire EU population, the six lines sometimes overlapped. For example, the graph displaying the six-item responses appeared as five lines for Spain because the two lines for “could not overcome your difficulties” and “constantly under strain” almost overlapped (Fig. 4).

Although responses to the six items essentially maintained a characteristic pattern across the 15 European countries, the graphs of the responses appeared to vary by country. For example, as indicated by the bidirectional arrows, for Italy and Greece, the probability of “score = 0” differed considerably for the six items, while they did not differ to that extent for Germany (Fig. 4).

**Figure 5:**
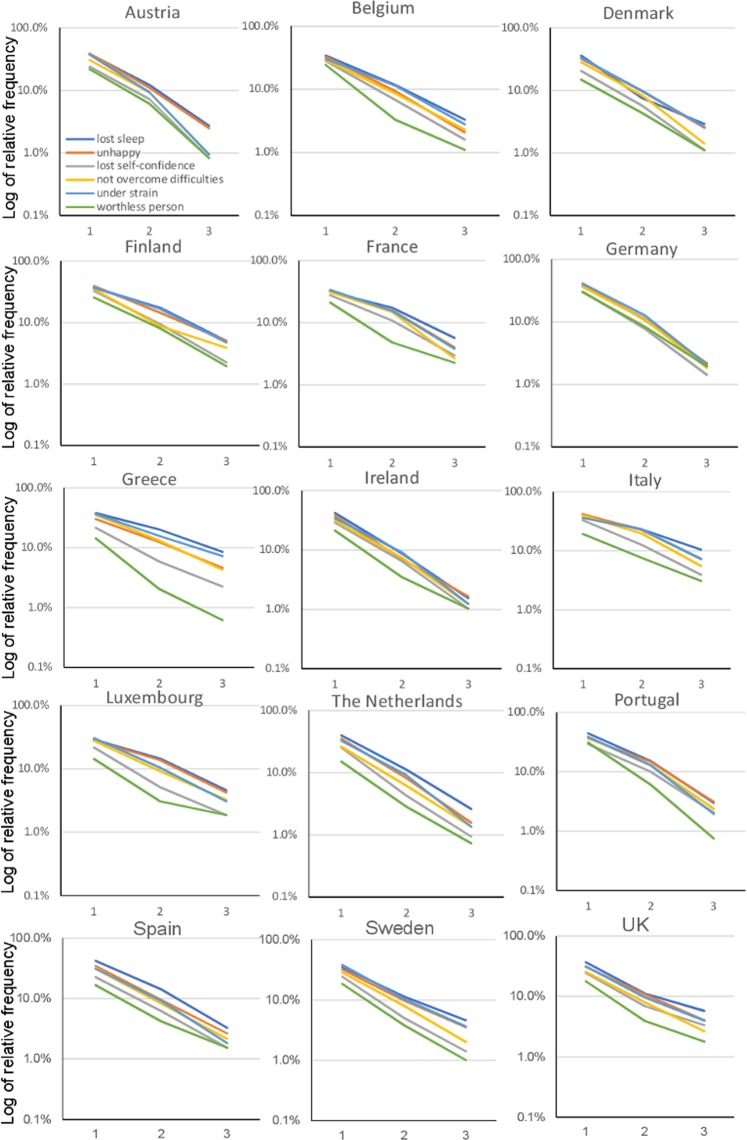
Using a logarithmic scale, the lines for the six negative items generally exhibit a parallel pattern from “score = 1” to “score = 3” across the 15 European countries. While the lines for Denmark, Germany, Ireland, the Netherlands, Spain, and the UK appeared to follow an almost parallel pattern, the lines for Greece did not follow a parallel pattern to that extent.

Using a logarithmic scale, the item response lines showed a parallel pattern between “score = 1” and “score = 3” across the 15 European countries (Fig. 5). The degree to which the six items followed a parallel pattern on a logarithmic scale differed by country. While the lines for Denmark, Germany, Ireland, the Netherlands, Spain, and the UK appeared to follow an almost parallel pattern, the lines for Greece did not follow a parallel pattern to that same extent (Fig. 5).

## Discussion

The results of this study showed that responses to the depressive symptom items in the GHQ-12 exhibited a characteristic pattern of item responses across the entire EU and in each country. The ratios between two adjacent response options, with the exception of the response option at the lower end, were similar across all six depressive symptom items. The item response pattern was characterized by intersections at a single point between “not at all = 0” and “no more than usual = 1” and parallel patterns from “no more than usual = 1” to “nearly every day = 3” on a logarithmic scale, consistent with the patterns observed in previous studies using other depression screening scales^10,12–14^. These results provide further evidence that responses to depression screening scales in the general population follow the same mathematical model regardless of the specific country.

There are some limitations to this study. First, as the similarity of the ratios between the two adjacent response options across all six items reflects the fit of the present model to the observed data^10^, we demonstrated that the ratios were similar across all six items. However, one major limitation of this procedure is that there is no unified method for interpreting the results. Thus, even after obtaining the results that the ratios were similar across all six items, we were unable to describe the degree of the present model’s fit using unified descriptors, such as “slightly,” “rather,” “well,” etc. Of note, as the present item response model is complicated, it is difficult to perform a unitary regression analysis using established distribution models (e.g., linear, normal, and geometrical). Further research is required to develop a method that describes how well the present model fits the observed data. Second, while the Eurobarometer questionnaire included six negative GHQ-12 items, it did not include the six positive GHQ-12 items. For this reason, we could not perform a pattern analysis of the responses to the six positive items. Previous studies using the CES-D data have reported that, unlike the depressive symptom items, the responses to the positive items do not exhibit a specific, common pattern^10,24^. Thus, it is necessary that further studies using the six positive GHQ-12 items be conducted.

Despite these limitations, the graphical analysis enabled us to detect a complicated pattern of the item responses, which is an advantage of this study. If the exact values of the item responses were only presented in a numerical table, we would not have been able to identify the complicated pattern of the item responses. In general, a graphical visualization is an indispensable tool for identifying the underlying, complicated patterns in empirical data^25–27^.

Across the entire EU population, the two lines for “unhappy and depressed” and “constantly under strain” appeared as one line because the two lines are close (Fig. 3). Furthermore, country-by-country analyses showed that the six lines often overlapped across each of the 15 EU countries (Fig. 4). The present item response model can explain these findings (Fig. 2). According to the model, if two different items have similar values for the probability of “score = 1,” the probabilities of “score = 0,” “score = 2,” and “score = 3” for the two different items are expected to be similar. In short, the finding that the two different lines often appear as one supports the theory that responses to depression symptom items follow a common pattern in a general population.

It remains unknown why responses to these items follow this characteristic pattern in the general population^10^. The response options on these scales usually start with the absence of symptoms at the lower end (e.g., “not at all”) and increase in symptom severity (e.g. “no more than usual,” “rather more than usual,” and “much more than usual”)^28^. Further research should focus on how the ratios of the adjacent higher severity response options are similar across all items. Previous studies have shown that, if the severity of depressive symptoms follows an exponential distribution in a general population and each of the higher severity response options cover the fixed proportion of their range, the pattern observed in the present and previous studies could emerge^29,30^.

In the present study, while the responses to the six items generally exhibited a characteristic pattern across the 15 European countries, the degree to which the six items followed this characteristic pattern varied by country. The lines for Denmark, German, Ireland, the Netherlands, Spain, and the UK appeared to exhibit almost parallel patterns on a logarithmic scale (Fig. 5). In a previous analysis of the CES-D data from the TILDA, the line for “insomnia” considerably diverged from the parallel pattern for other 15 depressive symptoms items^15^. Conversely, in the present study, the line for “lost much sleep over worry” in Ireland was almost parallel to the other five item lines (Fig. 5). This discrepancy may be explained by participants’ age profiles. While the Irish sample in Eurobarometer 56.1 comprised individuals of all generations aged ≥15 years (Mean age ± SD = 41 ± 18), the TILDA cohort comprised individuals aged 50 years or older^31^. As the prevalence of sleep problems increases with age^32^, the participants in the TILDA could exhibit more severe sleep disturbance than those predicted from the parallel pattern for the other 15 depressive symptoms CES-D items.

The present results showed that the lines of item responses on a logarithmic scale in Greece rather deviated from a parallel pattern (Fig. 5); in particular, the line for “thinking of yourself as a worthless person” did not follow a parallel pattern as that observed for the other items. The reason for this finding is unclear but may be related to the small sample sizes for scores higher than 2–3. In fact, the probability of “score = 3” for “thinking of yourself as a worthless person” in Greece was less than 1% (six individuals). Supporting the effect of sample size, the parallel pattern of item responses on a logarithmic scale appears to be more evident in the entire EU sample than in each country. Further studies with a large sample size are needed to confirm the reproducibility of the findings in Greece.

Finally, we come to the significance of the present findings. As noted in the introduction, statistical procedures that assume a normal distribution are currently used to evaluate the psychometric properties of depression screening scales. However, our findings provide further evidence that item responses on depression screening scales follow a non-normal distribution in a general population, suggesting that statistical procedures assuming normality require careful consideration when assessing these scales. Historically, there has been a dispute over whether parametric statistics can be applied to data with non-normal distributions. While a considerable number of researchers claim that parametric statistics are robust with respect to the violation of the assumption of normality, others maintain the logical view that parametric procedures cannot be applied. Further research is needed to determine whether parametric procedures such as analysis of variance, regression analysis, factor analysis, etc. can be applied to data using depressive symptom scales.

In conclusion, the results of this study suggest that the responses to the depressive symptom GHQ-12 items follow the same mathematical patterns across the general EU populations. These results suggest that the responses to depression screening scales follow a common characteristic pattern across the items regardless of the specific country. More research should be conducted to explore how responses to these items follow a common distribution pattern in a general population. The degree to which the present findings can be generalized to other areas of psychopathology or to various psychological conditions is unclear and warrants further examination.

## Method

### Dataset

The present study used data from Eurobarometer 56.1: Social Exclusion and Modernization of Pension Systems conducted in 2001^19^. Eurobarometer 56.1 focused on the social exclusion and modernization of pension systems, and the sample comprised 15,943 participants from 15 EU countries: Austria (n = 1000), Belgium (n = 1032), Denmark (n = 1001), Finland (n = 997), France (n = 1002), Germany (n = 2009), Greece (n = 1004), Ireland (n = 996), Italy (n = 992), Luxembourg (n = 600), the Netherlands (n = 1006), Portugal (n = 1001), Spain (n = 1000), Sweden (n = 1000), and the United Kingdom (n = 1303). The multistage probability sample of Europeans aged ≥ 15 years was based on each country’s total population and population density. Face-to-face interviews were conducted in participants’ homes. Eurobarometer 56.1 datasets are accessible to researchers worldwide through the Interuniversity Consortium for Political and Social Research (ICPSR)^19^. The participants’ socio-demographic characteristics have been described in detail elsewhere^19^.

### Ethics statement

This study is a secondary analysis of freely accessible public data. Our institutional ethics committees do not consider de-identified public data analysis as human research, and thus the need for ethical approval was waived.

### Measures

The Eurobarometer 56.1 questionnaire included the six negative GHQ-12 items. Each item assesses the frequency of symptoms experienced over the last few weeks on a four-point response scale [not at all = 0; no more than usual = 1; rather more than usual = 2; and much more than usual = 3]. The negative six items ask how often the participant has experienced the following: (1) lost much sleep over worry, (2) been feeling unhappy and depressed, (3) been losing confidence in yourself, (4) felt you could not overcome your difficulties, (5) felt constantly under strain, and (6) been thinking of yourself as a worthless person. The total score ranges from 0 to 18.

### Study sample

Individuals who did not respond to all six negative items (502 individuals, 3.1%) were excluded from the analysis. The final sample consisted of 15,441 individuals (male: 47.7%, mean age: 45 years; standard deviation 18 years): Austria (n = 963), Belgium (n = 1005), Denmark (n = 995), Finland (n = 978), France (n = 972), Germany (n = 1894), Greece (n = 984), Ireland (n = 972), Italy (n = 940), Luxembourg (n = 587), the Netherlands (n = 957), Portugal (n = 937), Spain (n = 981), Sweden (n = 991), and the United Kingdom (n = 1285). Detailed information about the demographic data for each European country is presented as Supplementary Data.

### Analysis

First, we analyzed the distributions of the responses to the six negative items across the entire EU population. We corrected the imbalance between the survey sample and the population using weighting adjustments. For international weighting, we applied the official population figures provided by Eurostat^33^. The percentage weight assigned to each nation is as follows: Austria (2.16%), Belgium (2.79%), Denmark (1.42%), Finland (1.35%), France (16.4%), Germany (20.28%), Greece (2.63%), Ireland (1.18%), Italy (14.81%), Luxembourg (0.15%), the Netherlands (4.19%), Portugal (2.52%), Spain (11.42%), Sweden (2.48%), and the United Kingdom (16.22%).

As noted above, if the ratio between two adjacent response options is similar across all six negative items, with the exception of the response option at the lower end, then all item response lines will show this characteristic pattern^11,14^. Thus, the ratios of “rather more than usual” to “no more than usual of the time” and of “much more than usual” to “rather more than usual” were calculated for all six items. Next, we graphically analyzed the patterns of the item responses. These response patterns for the six items across the entire EU population were visually presented on normal and logarithmic scales.

After confirming that the responses to the six items exhibited the same characteristic pattern in the entire EU population, which was consistent with previous reports^10,24^, country-by-country analyses were performed for the responses to the six negative items. The pattern of item response distributions across each country was analyzed by line graphs using normal and logarithmic scales. As the aim of this research corresponds to an exploratory data analysis for finding patterns, we did not conduct statistical hypothesis tests such as a comparison of the mean item scores across nations^27^. All analyses were performed using JMP Version 11.2.1 for Windows (SAS Institute, Inc., Cary, NC, USA).

### Ethics and consent to participate

The present study used de-identified data that is available to the public. The author’s institutional review board does not consider secondary analyses of publicly available data to be research on human subjects.

## Supplementary information

Dataset 1

## Data Availability

The datasets analyzed in the present study are available in the ICPSR repository, https://www.icpsr.umich.edu/icpsrweb/ICPSR/studies/3475.
